# Protective Effect of* Amphipterygium adstringens* Extract on Dextran Sulphate Sodium-Induced Ulcerative Colitis in Mice

**DOI:** 10.1155/2016/8543561

**Published:** 2016-08-21

**Authors:** Mario Rodriguez-Canales, Ruben Jimenez-Rivas, Maria Margarita Canales-Martinez, Ana Judith Garcia-Lopez, Nelly Rivera-Yañez, Oscar Nieto-Yañez, Yadira Ledesma-Soto, Luvia Enid Sanchez-Torres, Miriam Rodriguez-Sosa, Luis Ignacio Terrazas, Marco Aurelio Rodriguez-Monroy

**Affiliations:** ^1^Departamento de Inmunología, Escuela Nacional de Ciencias Biológicas, Instituto Politécnico Nacional, Prolongación de Carpio y Plan de Ayala s/n, 11400 Ciudad de México, Mexico; ^2^Laboratorio de Inmunobiología, Carrera de Medicina, FES Iztacala, UNAM, Avenida de los Barrios Número 1, Colonia Los Reyes Iztacala, 54090 Tlalnepantla, MEX, Mexico; ^3^Laboratorio de Farmacognosia, UBIPRO, FES Iztacala, UNAM, Avenida de los Barrios Número 1, Colonia Los Reyes Iztacala, 54090 Tlalnepantla, MEX, Mexico; ^4^Unidad de Biomedicina, FES Iztacala, UNAM, Avenida de los Barrios Número 1, Colonia Los Reyes Iztacala, 54090 Tlalnepantla, MEX, Mexico

## Abstract

*Amphipterygium adstringens *is an endemic species in Mexico commonly known as “cuachalalate.” Healers to treat gastritis, gastric ulcers, and gastrointestinal cancer have traditionally used the bark. We investigated the effects of alcoholic extract of* A. adstringens *(AaEE) in DSS-induced colitis in mice. The protective effect of AaEE was determined at 200 mg/kg by oral gavage for 10 days. We determine the effect of AaEE on clinical features (disease activity index), antioxidants, anti-inflammatory, and immunomodulatory activities in relation to the activity of SOD, CAT, and GPx, levels of proinflammatory cytokines, and changes both macroscopic and microscopic of the colonic mucosa. AaEE significantly reduced the inflammation of colon and significantly increased SOD and GPx activities. AaEE also significantly decreased TNF-*α*, IFN-*γ*, and IL-1*β* cytokine levels compared to DSS-treated mice and reduced both infiltration of inflammatory cells and the mucosal damage in colon. The results suggested the protective potential of AaEE in DSS-induced colitis and this might be attributed to its phytochemicals compounds that have been found to induce a wide spectrum of activities such as reduction in oxidative stress, suppression of inflammation, modulating numerous signal transduction pathways, and induction of apoptosis. The findings of this study suggest that AaEE has substantial potential for the treatment of inflammatory colitis.

## 1. Introduction

Ulcerative colitis (UC) is a major type of inflammatory bowel disease characterized by chronic, relapsing intestinal inflammation with extensive damage of colonic mucosa. It is presented by a variety of clinical manifestations, including attacks of abdominal cramps, pain, bloody diarrhea, bleed per rectum, weight loss, fever, and easy fatigability, which may begin gradually or start totally all at once [1, 2]. There are different drugs currently used in UC, including aminosalicylates, corticosteroids, immunosuppressants, or biological therapies such as the use of anti-TNF*α* antibodies. Although all of them have shown some grade of efficacy in these intestinal conditions, the frequency and severity of adverse effects, inconvenient dosing regimen, and partially prohibitive price limit their long-term use [3, 4]. For this reason, the development of new therapies that combine efficacy, convenient dosing, and fewer side effects is an important goal in human UC therapy. In this regard, the use of alternative therapies has emerged as a common approach in gastrointestinal diseases; actually, studies describe that almost half of IBD patients have ever taken or currently use complementary remedies [5, 6]. Different factors may contribute to this situation, including the lack of a complete response to standard therapy and the general feeling about a better safety profile of traditional remedies, in combination with the appreciation of an improved control of their disease. There are many different types of alternative and/or complementary therapies, although the botanical drugs are very relevant for the treatment of the intestinal inflammation. This can be mainly related to their safety, since they have been taken from ancient times, in addition to their reputed efficacy, most probably due to the presence of different active components that can concurrently target several pathways or mediators of the inflammatory response. However, most of these uses have an empirical basis, and in consequence, it is necessary to properly evaluate these botanical drugs to consider them as an adequate strategy to treat IBD [7]. Experimental colitis models have been used to identify therapeutic agents and elucidate the underlying physiologic mechanisms of UC. The widely employed DSS-induced colitis model recapitulates the histological characteristics of UC [8, 9].

*Amphipterygium adstringens *Schide ex Schlecht (Julianiaceae) is an endemic species in Mexico commonly known as “cuachalalate.” The bark has traditionally been used by healers to treat gastritis, gastric ulcers, gastrointestinal cancer, colic, fever, and also tooth pain. Moreover, anti-inflammatory, hypocholesterolemic, antifungal, and antiprotozoal activities have also been reported as properties for this plant [10–19].

Therefore, the present study aimed to investigate the antioxidant, anti-inflammatory, and immunomodulatory activities of* A. adstringens *ethanolic extract in a mouse model of experimental colitis induced by DSS, regarding the activity of superoxide dismutase (SOD), catalase (CAT), and glutathione peroxidase (GPx), cytokine levels, and macroscopic and microscopic changes of colonic mucosa.

## 2. Materials and Methods 

### 2.1. Reagents

 DSS (MW: 35,000–50,000; MP Biomedicals, Solon, OH, USA) was used for induction of colitis; SOD, CAT, and GPx kits were obtained from Cayman Chemical (Ann Arbor, MI, USA) and were used to determine the colonic antioxidant enzyme activities. Determination of cytokine levels was performed using a Bio-Plex Pro Mouse Cytokine 8-Plex Panel (Bio-Rad, Hercules, California USA). For antioxidant activity, 2,2-diphenyl-1-picrylhydrazyl solution (DPPH) was used.

### 2.2. Plant Material

The* A. adstringens* bark was collected in August 2013 in San Rafael, Coxcatlan, Puebla, and the botanical authentication of the specimen was done by M. C. Maria Edith Lopez Villafranco (curator at the IZTA Herbarium). Voucher specimens were deposited in the herbarium IZTA at the Facultad de Estudios Superiores Iztacala (voucher number IZTA-29285).

San Rafael is a village in the municipality of Coxcatlan, which is located southeast of the Tehuacan-Cuicatlan Valley at 18°12′ and 18°14′ North and 97°07′ and 97°09′ West, residing 957 m above sea level. The climate is dry or arid with summer rains and a mean temperature of 22°C [20]. The specimens were collected in the field with permission from the “Secretaria de Medio Ambiente y Recursos Naturales” (SGPA/DGVS/1266).

### 2.3. Mice

Female BALB/c mice 6–8 weeks of age were purchased from Harlan Laboratories (Mexico). Mice were maintained in a pathogen-free environment at the FES Iztacala, UNAM, animal facility according to Faculty Animal Care and Use Committee and government guidelines (official Mexican regulation NOM-062-ZOO-1999), which are in strict accordance with recommendations in the Guide for the Care and Use of Laboratory Animals of the National Institutes of Health (USA). Mice were sacrificed using CO_2_ chamber, and all efforts were made to minimize pain.

### 2.4. Preparation of* A. adstringens* Ethanolic Extract (AaEE)

The extract of the* A. adstringens* was obtained from dehydrated bark (555 g) through maceration with ethanol (2.0 L) at room temperature. After filtration, the solvent was evaporated under reduced pressure, generating the ethanol extract (AaEE). The yield of AaEE was 164.95 g (23.87%). 10 g of AaEE was dissolved in ethanol (100 mL) and hexane (100 mL) before being placed in a separatory funnel. After the solvent-solvent extraction, the hexane fraction (*F*_1_) was removed from the AaEE. To the ethanol residue was added chloroform (100 mL), after the solvent-solvent extraction, the chloroform fraction (*F*_2_), was removed. After removing the solvent, 0.078 g of hexane (0.78%) and 0.296 g of chloroform (2.96%) were obtained.

### 2.5. Experimental Colitis

Experimental colitis was induced by 4% DSS. Briefly, 6–8-week-old BALB/c mice were housed at a temperature of 22–24°C under a 12 h light/dark cycle with free access to water. After a 1-week period of adaptation, the mice were randomly divided into the following 4 groups (6 mice/group): untreated (no DSS), DSS-treated (4% DSS), DSS + AaEE (4% DSS and AaEE at 200 mg/kg body weight per day), and vehicle (100 *μ*L EOH 50% v/v). The untreated group received tap water without DSS. For the DSS-treated group and DSS + AaEE group, the water bottles of the mouse cages were filled with DSS 4%. The AaEE were orally administered for 10 days. The beginning of treatment and the colitis induction both were performed simultaneously.

The mice were scored daily with respect to body weight, stool formation, and bloody stool. The weight loss, stool formation, and bloody stool scores were averaged to determine the DAI. Scores were assigned as follows: weight change (0: <1%, 1: 1–5%, 2: 5–10%, 3: 10–15%, or 4: >15%), bloody stool (0: negative, 2: positive, or 4: gross bleeding), and stool formation (0: normal, 2: loose stool, or 4: diarrhea). On day 10, the mice were sacrificed by cardiac puncture under ketamine/xylazine anesthesia; blood was collected and centrifuged to isolate plasma, which was kept at −20°C until use for cytokine determination. Then, colon tissues were isolated.

### 2.6. Histology

Colon tissue samples were fixed in 10% formalin and embedded in paraffin. Then 5 micrometer-thick tissue sections were prepared and stained with hematoxylin and eosin (HE) to evaluate mucosal damage. The sections were also stained with Alcian blue to evaluate the presence of goblet cells.

### 2.7. Histopathological Analysis

Paraffin-embedded colon sections, fixed in formalin, were stained using hematoxylin and eosin (H&E). An examiner without prior knowledge of experimental procedures scored the degree of colitis. Assessment included noting of edema, extent of injury, leukocyte infiltration, crypt abscesses, and loss of goblet cells. In this grading system, inflammation severity was scored on a 0–3 scale (0: none; 1: slight; 2: moderate; 3: severe) as was the extent of injury (0: none; 1: mucosal; 2: mucosal and submucosal; 3: transmural); crypt damage was scored on a 0–3 scale (0: none; 1: basal third damaged; 2: basal two-thirds damaged; 3: loss of entire crypt and epithelium). At least 3 sections from each colon were analyzed to produce each score value. The scoring system yields minimal and maximal total scores of 0 and 9.

### 2.8. Cytokine Measurement

Determination of TNF-*α*, IFN-*γ*, IL-1*β*, and IL10 cytokine levels is using a Bio-Plex Pro Mouse Cytokine 8-Plex Panel (according to the manufacturer's protocol).

### 2.9. SOD, CAT, and GPx

Colon tissue samples were washed in 0.1 M phosphate buffer, pH 7.4. The excised colons were homogenized in phosphate buffer using a Bullet Blender homogenizer (Next Advance, NY, USA) and the homogenates were centrifuged at 10,000 ×g for 15 min at 4°C. The supernatant was collected for the biochemical estimations. The levels of SOD, GPx, and CAT activity were all determined using the methods provided by the assay kits (Cayman Chemical Company, USA). Cayman's SOD Assay Kit utilizes a tetrazolium salt for detection of superoxide radicals generated by xanthine oxidase and hypoxanthine. One unit of SOD is defined as the amount of enzyme needed to exhibit 50% dismutation of the superoxide radical. The SOD assay measures all three types of SOD (Cu/Zn, Mn, and FeSOD). Cayman's GPx Assay measures GPx activity indirectly by a coupled reaction with glutathione reductase (GR). Oxidized glutathione (GSSG), produced upon reduction of hydroperoxide by GPx, is recycled to its reduced state by GR and NADPH. The oxidation of NADPH to NADP+ is accompanied by a decrease in absorbance at 340 nm. Under conditions in which the GPx activity is rate limiting, the rate of decrease in the A_340_ is directly proportional to the GPx activity in the sample. Cayman's CAT Assay Kit utilizes the peroxidatic function of CAT for determination of enzyme activity. The method is based on the reaction of the enzyme with methanol in the presence of an optimal concentration of H_2_O_2_. The formaldehyde produced is measured colorimetrically with 4-amino-3-hydrazino-5-mercapto-1,2,4-triazole (Purpald) as the chromogen. Purpald specifically forms a bicyclic heterocycle with aldehydes, which upon oxidation changes from colorless to a purple color.

### 2.10. Antioxidant Activity (DPPH Decoloration Assay)

The antioxidant activity was determined according to the method by Okusa et al. [21]. Ninety-six-well ELISA plates were filled with extract concentrations ranging from 1 to 100 *μ*g/mL, HPLC grade methanol served as a blank sample, and a 2,2-diphenyl-1-picrylhydrazyl solution (DPPH) (100 *μ*M) served as a control. The plates were incubated for 30 min at 37°C, and the absorbance values were determined at 517 nm with an ELISA plate reader. The antioxidant activity values were determined according to the following equation: %  inhibition = [(absorbance of control − absorbance of sample)/absorbance of control] − 100. The concentration leading to 50% inhibition (SC_50_) was determined graphically. Quercetin was used as a reference (positive control).

### 2.11. Total Phenolic Content

The Folin-Ciocalteu method [22] was used to determine the total phenolic content of a 0.05 mg/mL concentration of the methanolic extract. Distilled water (6 mL) and 500 *μ*L of Folin-Ciocalteu reagent were added, and after 5 minutes, 1.5 mL of Na_2_CO_3_ (200 g/L) and distilled water was added. Methanol was used as the blank sample. After incubation at room temperature for 2 hours, absorbance readings were measured using a spectrophotometer at 760 nm. The mean of three readings was used to interpolate with the gallic acid curve (6.25, 12.5, 25, 50, 100, and 200 *μ*g/mL), and the total phenolic content was expressed in mg of gallic acid equivalents (GAE)/g of extract.

### 2.12. Total Flavonoid Content

The Dowd method [23] was used to determine the flavonoid content. A solution of 2% aluminum trichloride (AlCl_3_) in HPLC grade methanol and an extract concentration of 0.2 mg/mL was used. Readings at 415 nm using spectrophotometer were taken after 10 minutes against a blank sample (5 mL extract solution and 5 mL methanol without AlCl_3_). A quercetin (1–100 *μ*g/mL) calibration curve was used as the standard. The mean of three readings was expressed as mg of quercetin equivalent QE/g of extract.

### 2.13. Analysis of the Chemical Composition by HPLC and GC-MS

The AaEE was loaded onto the HPLC HP Series 1100 separations module from Hewlett-Packard (Wilmington, DE, USA), equipped with an Allsphere ODS-1 column, at 269 bar pressure and a temperature range of 22°C-23°C. The mobile phase consisted of methanol : acetonitrile : H_2_O (25 : 25 : 50) for 20 minutes. A DAD detector was used at a wavelength of 260 nm with a full scan of 200–400 nm.

*F*_1_ and *F*_2_ fractions were injected into a gas chromatograph 6850 (China) equipped with a RTX column (30 m × 0.25 mm i.d., film thickness, 0.25 *μ*m). The temperature of the column was programmed starting at 70°C for 2 min, and then the temperature was increased 8°C/min to 270°C. At 270°C, a programmed linear gradient increased the temperature 10°C/min to 290°C. The injector and detector temperatures were 250°C and 290°C, respectively. The gas carrier was helium at a flow rate of 0.9 mL/min. The peak areas were measured by electronic integration. The relative amounts of the individual components were based on the peak areas. GC-MS analysis was performed on a AGILENT 5975C (China) mass spectrometer. The mass spectra were recorded at 70 eV. *F*_1_ and *F*_2_ fractions components were identified by comparison of the retention times and the mass spectra with the NIST/EPA/NIH Mass Spectral Library.

### 2.14. Statistical Analysis

Results are expressed as mean values ± SD. Statistical analysis was performed using one-way ANOVA. *P* value < 0.05 was considered significant. Calculations were performed using GraphPad Prism (version 6.0; GraphPad Software Inc., San Diego, CA, USA).

## 3. Results

### 3.1. AaEE Increased the Survival Rate in DSS-Induced Colitis Mice

**Figure 1:**
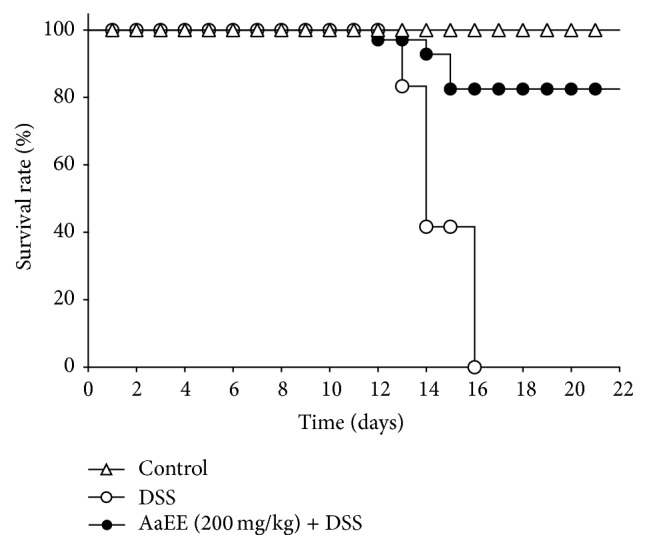
Effect of AaEE on the survival ratio of mice. AaEE prolonged animal survival *P* < 0.001 versus DSS (Mantel-Cox test).

Body weight loss and bloody stools were observed in mice acutely exposed to DSS. These symptoms were more marked at the end of the experimental period, when animals showed multiple clinical signs of severe disease, including marked weight loss (more than 15%). The mortality in the DSS group started at day 13, (16.67%); at day 16, 100% of mortality was registered. However, survival curves showed that AaEE significantly increased the survival rate of the mice (Figure 1).

### 3.2. Treatment with AaEE Inhibit Body Weight Loss, Severity of DAI, and Shortened Colon Length in Colitic Mice

**Figure 2:**
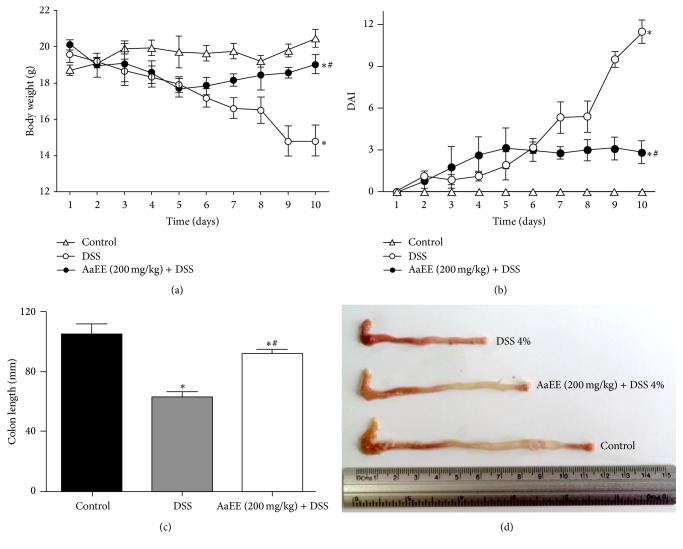
AaEE attenuates clinical signs in mice with DSS-induced colitis. (a) Changes in the body weights and (b) clinical scores of AaEE-treated mice and control mice given 4% DSS were monitored every day. (c) Colons were obtained from mice 10 days after commencement of DSS administration, and their lengths were measured. (d) Macroscopic features of the colons. Control group (without DSS), 4% DSS administration group (DSS), and 4% DSS with V at 200 mg/kg body weight per day. Data shown are from 3 independent experiments and are expressed as mean ± SD (*n* = 6 per group). ^*∗*^ *P* < 0.05, versus control; ^#^ *P* < 0.05, versus DSS.

The main clinical manifestations produced by the model of acute DSS colitis are diarrhea and blood in the stool, always accompanied by considerable weight loss. In the DSS-treated group, a loss of body weight is recorded between days 3 and 4 after administration of DSS (4%) and this loss remained significantly higher compared with the group of normal mice until day 10 (*P* < 0.05). Treatment with AaEE group (200 mg/kg) + DSS reversed the weight loss starting from day 6 (Figure 2(a), *P* < 0.05). DAI scores significantly increased up between days 6 and 7 in DSS group, as indicated by the incidence of diarrhea, weight loss, and bloody stools. However, AaEE-treated group exhibited significantly attenuated DSS-induced disease severity (Figure 2(b)). In addition, we found that the colon was significantly shorter in the DSS-treated mice (62.80 ± 4.25 mm) than in the control group (104.9 ± 7.56 mm). Interestingly, treatment with AaEE prevented colon shortening significantly (*P* < 0.05). The colon length was 92.73 ± 2.71 mm in these mice (Figures 2(c) and 2(d)).

### 3.3. AaEE Reduced Histopathological Damage in DSS-Induced Ulcerative Colitis in Mice

**Figure 3:**
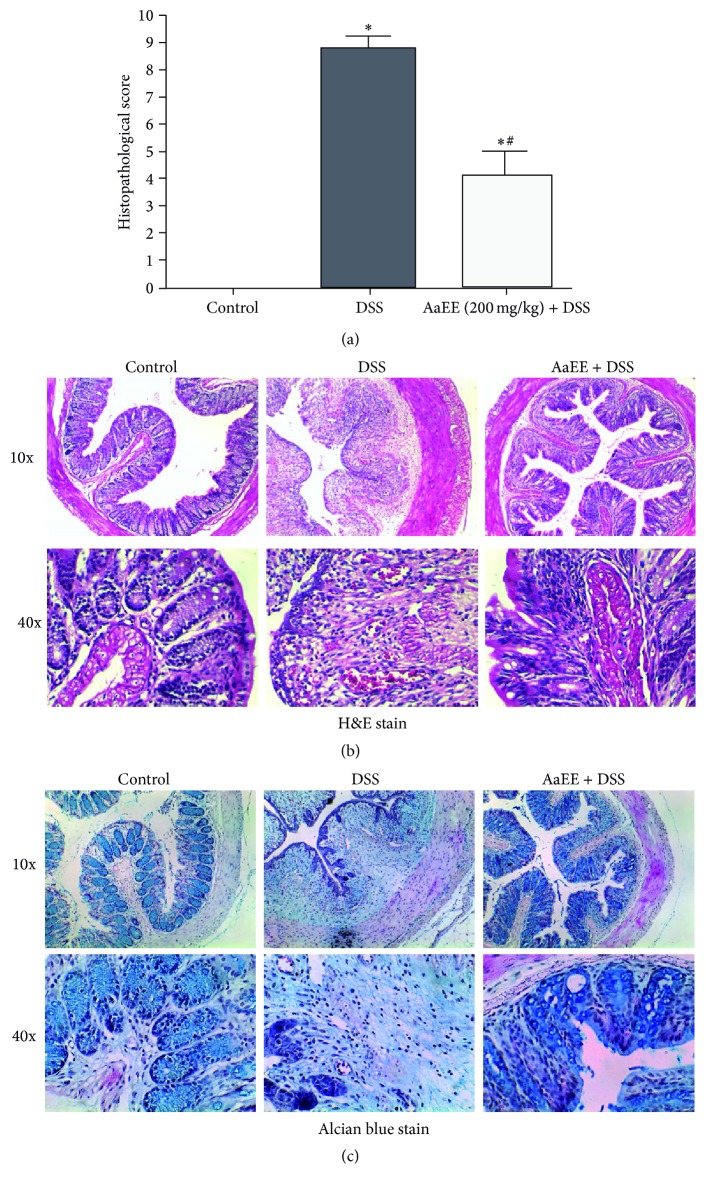
AaEE treatment improves the histopathological score in DSS-induced colitis mice. Mice colons were obtained at 10 days after DSS administration and were stained with H&E and Alcian blue. H&E and Alcian blue staining were performed on colon sections obtained from mice belonging to the control group (no DSS), DSS-treated (4% DSS), and AaEE (200 mg/kg) + DSS. Histopathological scores were analyzed from slides (a). Data from 3 independent experiments is shown and expressed as the mean ± SD (*n* = 6 per group). ^*∗*^ *P* < 0.05 versus control; ^#^ *P* < 0.05 versus DSS. Images were obtained at 10 and 40x magnification (b and c).

Histological examination of H&E-stained colonic sections under light microscopy revealed that DSS mice showed typical inflammatory changes in colonic architecture, such as ulceration, crypt dilation, loss of tissue architecture, and goblet cell depletion, as well as cell infiltration; the damage was assessed from the colonic histopathological scores (range 0–9). Compared to control mice, DSS mice showed significantly increased histopathological scoring of disease (Figure 3(a)). After treatment with AaEE (200 mg/kg), the ulcer area was significantly reduced, and edema and adhesion were alleviated. The inflammatory cells were less prone to infiltration and were localized in the mucosa (Figure 3(b)).

### 3.4. Colonic Antioxidant Enzyme Activities of AaEE

**Figure 4:**
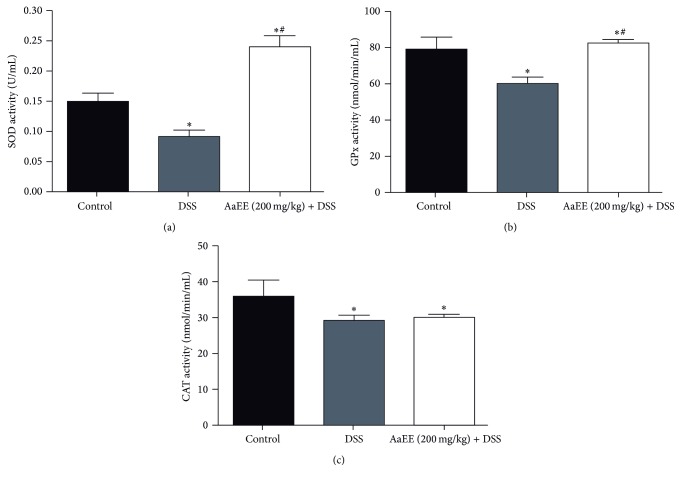
Protection of AaEE against oxidative stress in colorectums during ulcerative colitis induced by DSS in mice. SOD activity (a), GPx activity (b), and CAT activity (c). Data represents as mean ± SD (*n* = 6), ^*∗*^ *P* < 0.05 versus control group; ^#^ *P* < 0.05 versus DSS group.

The activity of antioxidant enzymes in colonic tissues of control and experimental group of mice is shown in Figure 4. A significant (*P* < 0.05) decrease in the activities of SOD, GPx, and CAT was evident in DSS-induced mice when compared with untreated mice. In contrast, AaEE-treated mice exhibited more prominent activities of the antioxidant enzymes SOD and GPx (0.24 ± 0.019 U/mL and 82.21 ± 2.81 nmol/min/mL, resp.) as compared with DSS group. CAT showed no significant difference between the group treated with DSS and AaEE + DSS group

### 3.5. AaEE Treatment Inhibited Proinflammatory Cytokine Production during Colitis

**Figure 5:**
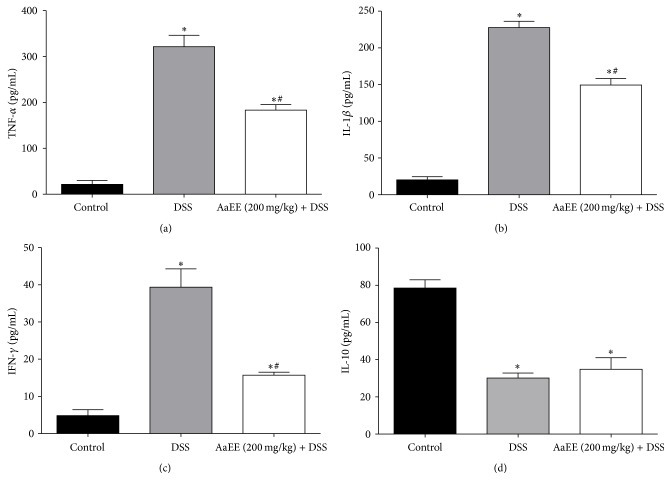
Effect of AaEE on cytokines in DSS-induced colitis. TNF-*α* (a), IFN-*γ* (b), IL-1*β* (c), and IL-10 (d) concentration in the plasma of mice from all groups. AaEE reduces the systemic levels of TNF-*α*, IFN-*γ*, and IL-1*β* and there were no significant differences on the levels reported for the IL-10 between the DSS group and the group of AaEE. All results are expressed as the mean ± SD. ^*∗*^ *P* < 0.05 versus control; ^#^ *P* < 0.05 versus DSS group.

The effects of AaEE on serum levels of several proinflammatory cytokines such as TNF-*α*, IFN-*γ*, and IL-1*β* were evaluated by Bio-Plex Pro Mouse Cytokine assay. As shown in Figure 5(a), DSS treatment increased significantly the serum TNF-*α* levels to 321.5 ± 24.75 pg/mL, compared to the control group (21.62 ± 8.44 pg/mL). However, AaEE treatment significantly reduced the levels observed in the DSS group to 183.3 ± 12.02 pg/mL (*P* < 0.05). With respect to IFN-*γ* both treatment groups AaEE and DSS showed high levels of this cytokine compared to control group (4.82 ± 1.6 pg/mL). However AaEE group displayed significantly lower levels of IFN-*γ* than DSS mice (15.69 ± 0.81 pg/mL and 39.36 ± 4.9 pg/mL, resp.) (*P* < 0.05) (Figure 5(b)). For DSS group, serum level of IL-1*β* increased more than ten times (227.6 ± 8.66 pg/mL versus normal controls 20.15 ± 4.35 pg/mL). However, treatment with AaEE significantly reduced IL-1*β* observed in the DSS group to 149.4 ± 8.84 pg/mL (*P* < 0.05) (Figure 5(c)). Systemic levels of IL-10 were increased in the AaEE-treated group in comparison with the DSS group, although no statistically significant differences were observed between these groups (Figure 5(d)).

### 3.6. Total Polyphenolic (TPC), Flavonoid Content (TFC), and Antioxidant Capacity (SC_50_)

The total polyphenolic content (TPC) of AaEE was determined by Folin-Ciocalteu assay. According to literature, phenols are the main responsible compounds of the antioxidant activity [24]; for this reason, the AaEE has been analyzed. Results of AaEE were expressed as mg of gallic acid equivalent (GAE)/g of extract (Table 1), by using a standard curve. AaEE displayed a TPC of 276.0 mg EAG/g. Total flavonoid content was expressed as mg of quercetin equivalent (QE)/g of dried extract (Table 1). AaEE showed a content of flavonoids of 49.4 mg QE/g.

With respect to the antioxidant capacity, generally, phenolic compounds are responsible for biological activities as antioxidant capacity and inhibition of enzymes involved in common diseases. In vitro antioxidant tests using free radical traps are relatively straightforward to perform. Among free radical scavenging methods, the one involving 2,2-diphenyl-1-picrylhydrazyl (DPPH) is rapid, simple, highly reproducible, and inexpensive in comparison to other test models. Results were expressed as SC_50_ (Table 1). According to Al-Fatimi et al. [25], AaEE showed a good DPPH scavenging activity (SC_50_ of 36.04 *μ*g/mL), while quercetin (used as standard) showed SC_50_ of 4.6 *μ*g/mL (Table 1).

### 3.7. Chemical Characterization of the Extract by HPLC and CG-EM

By HPLC were identified 4 compounds (Table 2) and 15 by GC-MS (Table 3).

## 4. Discussion

The standard treatments for colitis are generally immunosuppressant and anti- inflammatory drugs, which have many undesirable side effects. Therefore, better therapeutic agents that effectively attenuate mucosal inflammation with minimum or no side effects are needed. Plant-based, traditional remedies are now being used as alternative therapeutic strategies. Natural products, herbs, and dietary components have shown therapeutic effect against inflammatory colitis [3, 7]. Medicinal plant-based traditional medicine has long provided ethnopharmacotherapy for the treatment of chronic inflammatory disorders. The pharmacological activity of medicinal plants appeared to correlate with the presence of active compounds having antioxidant and anti-inflammatory activities. Several studies reported that* Amphipterygium adstringens *commonly known as “cuachalalate” exhibit various biological activities, including antioxidant, anticancer, and anti-inflammatory properties [10–19]. With this purpose, the present study has evaluated for the first time the potential application of ethanolic extract from* A. adstringens *(AaEE) in the DSS-induced colitis model in mice.

In this study, we showed that AaEE inhibited experimental colitis, resulting in overall attenuation of inflammation DAI, including colon length and body weight changes. The effects of AaEE were further confirmed by the H&E staining histological characterization in mouse acute colitis and colon tissue. The oral administration of 4% DSS in mice causes acute colitis. Animals from this group showed a variety of clinical symptoms of IBD including diarrhea, bloody stool, and weight loss. A Disease Activity Index score was used as a reliable tool to assess the extent of the gastrointestinal disease in the DSS-induced experimental colitis. At the end of the experimental period, the clinical symptoms were more pronounced. Our observations are in agreement with those reported in other studies using the DSS-induced colitis model [2, 26]. However, mice treated with AaEE (200 mg/Kg) significantly improved the clinical score, suggesting a protective effect of AaEE in this model of colitis. The histological scores indicate that treatment with AaEE decreased mucosal damage characterized by loss of crypt glands and epithelium destruction and inhibited colon shortening as observed in the DSS-alone group. Moreover, the Alcian blue staining showed regular mucosal structure with goblet cells secreting mucus in the AaEE + DSS group, in stark contrast to the DSS-treated mice. This observation suggests that* A. adstringens* treatment may protect mice from DSS-induced colitis by promoting mucus secretion.

Oxidative stress is one of the most crucial factors causing UC. Oxidative stress is known to damage cellular macromolecules such as DNA, lipids, and proteins. In the present study, the activity of enzyme antioxidants such as SOD, CAT, and GPx was decreased in DSS-induced mice. It has been proposed that an imbalance between prooxidant and antioxidant mechanisms may play an important role in the development of intestinal inflammation and mucosal tissue injury in colitis [27]. SOD plays an important role in protecting cells from oxidative damage by converting O^•−^ into H_2_O_2_. CAT and GPx then transform the generated H_2_O_2_ into H_2_O, thus preventing the harmful effects of oxidative radicals and the initiation processes of lipid peroxidation. It has been shown that SOD, GPx, and CAT levels are decreased in chemically induced colitis [28]. In agreement with this, our results also revealed a decrease of SOD, CAT, and GPx activities in colonic mucosa of the inflamed control animals, while the levels of both SOD and GPx were significantly increased in mice treated with AaEE (200 mg/Kg). CAT activity was slightly higher in the AaEE + DSS group than in the DSS group, although the difference was not significant; it has been reported that there is an increase in CAT activity by administering natural products as treatments against UC and this appears to be due to the fact that CAT has a lower affinity for that ROS comparing to SOD and GPx. This result comes in concordance with many previous studies that reported that GPx plays a much greater role in the removal of H_2_O_2_ than CAT [29–31]. Therefore, our findings indicate that AaEE treatment of colitis might be reducing the extent of colonic injury by its antioxidant ability.

On the other hand, inflammatory responses play a critical role in the pathogenesis of UC. The increased proinflammatory cytokines such as TNF-*α*, IFN-*γ*, and IL-1*β* amplify the inflammatory cascade and result in intestinal tissue damage in UC induced by DSS [32]. Among those cytokines, the overexpression of TNF-*α* is vital in intestinal mucosal impairment [33]. Adalimumab, a TNF-*α* blocker, has been successfully used for the treatment of IBD patients in the clinic [34]. In addition, IL-1*β* is a key mediator of the progression of UC. Inhibition the action of IL-1*β* can attenuate the severity of diarrhea and reduce infiltration of inflammatory cells into the intestinal tissue [35]. In our study, the levels of TNF-*α*, IFN-*γ*, and IL-1*β* were remarkably reduced by AaEE in DSS-induced colitis mice, suggesting that the protective effect of AaEE against colonic injury is related to the downregulation of TNF-*α*, IFN-*γ*, and IL-1*β*. With respect to IL-10, this cytokine contributes to the differentiation of regulatory T cells (Treg), while suppressing dendritic cell-associated Th1 and Th17 immunity, as well as regulating inflammatory responses [36, 37]. However, the fact that IL-10 levels did not show significant changes in our experiments is indicative that downmodulation of the inflammatory response by AaEE treatment is not associated with IL-10 levels and perhaps some of the compounds identified in our extracts of* A. adstringens* may act directly in the modulation of such inflammatory cytokines which are key to generate this disease. It would be worth testing whether these extracts act by blocking some of the intracellular pathways associated with inflammatory responses such as the JAK/STAT pathway or NFkB activation.

The present study was specially focused on the effects of AaEE on DSS-induced colitis. It is demonstrated here for the first time that oral administration of AaEE effectively attenuates colonic inflammation in mice. These results clearly demonstrate that, in a mouse model of colitis, body weight loss, DAI, colon length, and histological score were significantly reduced after 10 days of AaEE treatment (200 mg/kg day). Furthermore, AaEE attenuated the DSS-induced levels of TNF-*α*, INF-*γ*, and IL-1*β* and also increased the SOD, CAT, and GPx activities in colonic mucosa of mice treated with AaEE. These results give some clues about the compounds involved in the intestinal anti-inflammatory effect of the* A. adstringens* extract. One of these mechanisms could be related to the well-known antioxidant properties ascribed to the majority of the plant extracts, due to their content in polyphenolic compounds, as it happens with the present extract that contains flavonoids, which has been reported to exert antioxidant properties [38, 39]. For example, the chemical structure of catechin consists of a polyphenolic ring condensed with six-member oxygen containing heterocyclic ring that carries another polyphenolic ring. The 3′,4′ catechol structure on the polyphenolic ring is a potent scavenger of peroxyl, superoxide, and peroxy nitrite radicals. The presence of hydroxyl groups in the structure also enhances the inhibition of lipid peroxidation [40, 41]. Epigallocatechin-3-gallate (EGCG), a catechin of the green tea plant* Camellia sinensis*, shows strong expanding beneficial effects in studies of diabetes, Parkinson's disease, Alzheimer's disease, stroke, inflammation, obesity, and cancer [42]. EGCG exerts its antioxidant activity via decreasing NO and malondialdehyde (MDA) and increasing SOD and ameliorates mucosal inflammation by inhibiting the production of TNF-*α*, IFN-*γ*, and NF-*κ*B. EGCG reduces experimental colon injury by inhibiting macrophage chemotaxis toward N-formyl-L-leucyl-L-phenylalanine, thereby suppressing the mast cells and macrophage activities [43]. Naringenin is another important flavonoids present in AaEE. Several studies revealed its pharmacological effects including antidiabetic, antiatherogenic, antidepressant, immunomodulatory, antitumor, hypolipidemic, antioxidant, and anti-inflammatory potentials [44]. Naringenin abrogated experimental colitis by downregulating proinflammatory mediators like iNOS, ICAM-1, monocyte chemoattractant protein-1, COX-2, TNF-*α*, and IL-6. It also decreased mucosal Toll-like receptor 4 and NF-*κ*B p65 expression, thereby providing evidence for its potential in colitis [45]. In another study, naringenin ameliorated DSS-induced colitis by restoring the expression of tight junction proteins such as occludin, junctional adhesion molecule-A, and claudin-3 [46]. Another important group of compounds present in the* A. adstringens* extract are the fatty acids; they have been useful in numerous diseases like arthritis, diabetes, obesity, asthma, atherosclerosis, and cancer [47]. *α*-Linolenic acid has been reported to have cardiovascular-protective, anticancer, neuroprotective, antiosteoporotic, antioxidative, and anti-inflammatory effects [48]. In a model of TNBS-induced colitis, supplementation of *α*-linolenic acid in diet restored the 8-isoprostanes, GSH levels, and iNOS expression. It protected against colonic inflammation by reducing TNF-*α* secretion, NF-*κ*B activation, LTB4, and COX-2 expression [49]. In another study, *α*-linolenic acid decreased the expression of adhesion molecules like ICAM-1, VCAM-1, and VEGFR-2, providing avenues for designing diets beneficial to IBD [50].

## 5. Conclusion

These phytochemicals compounds of AaEE have been found to induce a wide spectrum of activities such as reduction in oxidative stress, suppression of inflammation, and cell proliferation and modulating numerous signal transduction pathways. The findings of this study suggest that the extract of* A. adstringens* (“cuachalalate”) has substantial potential for the treatment of inflammatory colitis.

## Figures and Tables

**Table 1:** Results of total polyphenol content (TPC) and total flavonoid content (TFC) and antioxidant capacity (SC) of AaEE.

	TPC mg (GAE/g)	TFC (mg QE/g)	DPPH (SC_50_ *µ*g/mL)
AaEE	276.0	49.4	36.04
Quercetin	—	—	4.6

(GAE)/g = mg gallic acid equivalent per gram of dried extract; (QE)/g = mg of quercetin equivalent per gram of dried extract; SC = scavenging capacity.

**Table 2:** HPLC analysis of AaEE.

Rt (min)	Name	Type
3.37	Catechin	Flavonoid
4.42	Catechol	Phenol
9.28	Naringin	Flavonoid
10.11	Pinocembrin	Flavonoid

Rt: retention time.

**Table 3:** GC-MS analysis of *F*_1_ and *F*_2_ fractions.

Rt (min)	Name	Type
8.720	*α*-Terpineol	Monoterpene
13.813	Myristic acid, ethyl ester	Fatty acid
14.532	Pentadecanoic acid, methyl ester	Fatty acid
14.763	Palmitic acid, methyl ester	Fatty acid
15.013	Palmitic acid	Fatty acid
15.744	Palmitelaidic acid, ethyl ester	Fatty acid
15.918	Linoleic acid	Fatty acid
15.956	Oleic acid, methyl ester	Fatty acid
16.091	Hexadecanoic acid, 14-methyl-, methyl ester	Fatty acid
16.334	Linoleic acid, ethyl ester	Fatty acid
16.367	Oleic acid, ethyl ester	Fatty acid
16.501	Stearic acid, ethyl ester	Fatty acid
16.373	Glyceryl monooleate	Fatty acid
16.405	Ethyl 9-hexadecenoate	Fatty acid
18.780	Cardanol	Phenol

Rt: retention time.
